# A cadaveric study of the testicular artery and its clinical significance

**DOI:** 10.1590/1677-5449.007516

**Published:** 2016

**Authors:** Sushma R. Kotian, Arvind Kumar Pandey, Supriya Padmashali, Judith Jaison, Sneha Guruprasad Kalthur

**Affiliations:** 1 Manipal University, Kasturba Medical College, Department of Anatomy, Manipal, Karnataka, India.

**Keywords:** testicular artery, abdominal aorta, renal artery, suprarenal artery, artéria testicular, aorta abdominal, artéria renal, artéria suprarrenal

## Abstract

**Background:**

Knowledge of testicular artery variations is vital to ensure that they are not neglected during a variety of different operative techniques, since damage can cause testicular atrophy.

**Objectives:**

The present study was therefore intended to identify variants in the origin and course of the testicular arteries. An attempt was made to classify the arteries based on their various origins.

**Methods:**

This study examined 42 formalin-fixed cadavers of 40 to 70-year-old adult males. Variant origins of the testicular artery were identified and classified. Variations in the origin and course of the artery were colored, photographed, and documented. The distances between the origins of the testicular arteries and the mid-points of the origins of the renal arteries were measured.

**Results:**

Testicular arteries were classified into four categories on the basis of origin. This variability was defined in relation to the renal and inferior mesenteric arteries. The mean distance between the origin of the testicular artery and the mid-point of the origin of the renal artery were 3.08 and 3.47 cm, on the right and left sides respectively. Variations were almost exclusively found on the left side. The variations observed included multiple arterial twigs forming the testicular artery, suprarenal arteries arising from the testicular artery, and testicular artery duplication.

**Conclusion:**

This study provides an insight into variations in the testicular artery and proposes a classification which could help surgeons during a variety of procedures on the male abdomen and pelvis.

## Resumo

**Contexto:**

O conhecimento de variações da artéria testicular é vital para assegurar que essas artérias não serão negligenciadas durante a realização de diferentes técnicas operatórias, já que qualquer dano poderia causar atrofia testicular.

**Objetivos:**

Este estudo teve como objetivo identificar variações na origem e no trajeto das artérias testiculares. Foi feita uma tentativa de classificar as artérias com base em suas diferentes origens.

**Métodos:**

Este estudo examinou 42 cadáveres de adultos do sexo masculino com idade entre 40 e 70 anos preservados em formol. As diferentes origens da artéria testicular foram identificadas e classificadas. Variações na origem e no trajeto da artérias foram coloridas, fotografadas e documentadas. Foram medidas as distâncias entre a origem das artérias testiculares e o ponto médio da origem das artérias renais.

**Resultados:**

As artérias testiculares foram classificadas em quatro categorias com base em sua origem. Essa variabilidade foi definida com relação às artérias renal e mesentérica inferior. A distância média entre a origem da artéria testicular e o ponto médio da origem da artéria renal foi de 3,08 e 3,47 cm nos lados direito e esquerdo, respectivamente. As variações foram encontradas quase exclusivamente no lado esquerdo. As variações observadas incluíram múltiplos ramúsculos formando a artéria testicular, artérias suprarrenais surgindo a partir da artéria testicular, e duplicação da artéria testicular.

**Conclusão:**

Este estudo traz informações sobre variações da artéria testicular e propõe uma classificação que poderia ajudar os cirurgiões durante diferentes procedimentos realizados no abdome e na pelve de pacientes do sexo masculino.

## INTRODUCTION

Adequate knowledge about anatomical variations in the origin, course, and distribution of vessels is crucial when performing endovascular diagnostic or curative surgeries in the abdomen and pelvis. The male gonadal arteries, i.e., the testicular arteries (TAs) are one of the paired vessels arising from the anterolateral aspect of the abdominal aorta (AA), usually inferior to the level of the origin of the renal artery (RA). Although variations in the position of the origin of the TAs are rare, they can also arise from the renal, accessory renal, middle suprarenal, inferior phrenic, lumbar, or even common iliac or internal iliac arteries. Occasionally, the TAs may even be absent. In such cases, the gonads receive oxygenated blood via vesical/prostatic arteries.^1^^,^ 2

Each TA is long and slender and courses inferolaterally under cover of the parietal peritoneum. The right-most artery often lies in front of the inferior vena cava. The artery may also be doubled, tripled, or even quadrupled entirely or along some portion of its course.^2^ The corresponding veins accompany the testicular arteries throughout their course. These vessels cross the psoas major muscle and later traverse the deep inguinal ring to constitute the spermatic cord.^1^

Information about the origin and course of the TA is surgically significant since undue ligature during invasive procedures can cause testicular atrophy. Furthermore, unusual course and location of the TA are important in many different surgical procedures that involve it.^3^ Surgeons need to understand the morphologic variations of these arteries and ensure that they are not neglected, compromising oxygenation of the gonads.

Previous studies have focused on variations of the TAs,^4^^-^ 15 but a classification of the TAs based on variability of their level of origin is seldom available in the existing literature.

It has been stated in the literature that the TA is situated a little inferior to the RA.^1^ However, the exact distance between them has not been explored.

Knowledge of variability of the testicular artery is most important for clinicians performing procedures like renal transplantation, interventional radiologic procedures, and renal vascular surgeries.

Therefore, the present study was designed to look into the possible variations of the origin and course of the TA. An attempt was made to classify the artery based on its variable origin. Additionally, the distance between the origins of the TA and the RA was also explored.

## METHODS

The present study was conducted on 42 formalin-fixed cadavers of adult males aged from 40 to 70 years.

The abdomen and posterior abdominal wall of all cadavers were dissected to identify variations in the origin and course of the TA. The AA was cleaned along its entire length. The connective tissue surrounding the great vessels and the nerve plexus around it were removed to provide a better view of their branches and their origins. The coils of the intestine and lymph nodes were detached to provide a clear view. The TA was identified and neatly dissected along its course from the point of its origin up to the deep inguinal ring from which point it becomes a component of the spermatic cord.

Variations in the origin and course of the TA were identified, colored, and photographed. Each TA was classified on the basis of the variability of its origin. The distance between the origin of the TA and the mid-point of the origin of the RA was measured using a digital Vernier caliper.

## RESULTS

**Figure 1:**
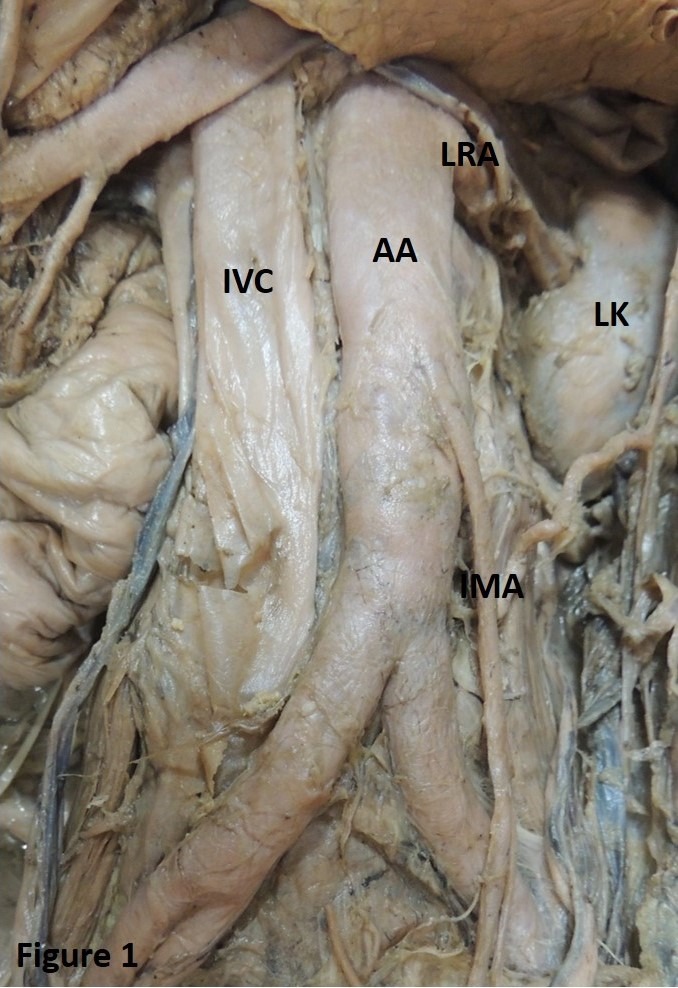
Showing absence of the testicular artery. AA: abdominal aorta; IMA: inferior mesenteric artery; IVC: inferior vena cava; LK: left kidney; LRA: left renal artery.

**Figure 2:**
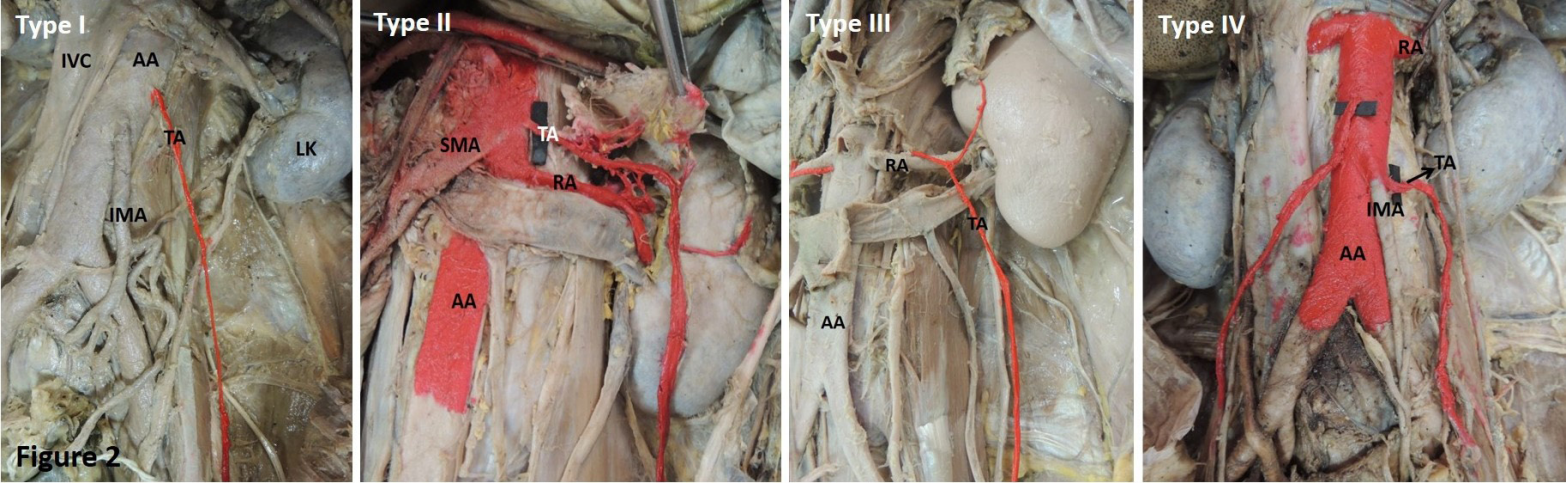
Representative images of the classification of the testicular artery TA based on its origin. AA: abdominal aorta; IMA: inferior mesenteric artery; IVC: inferior vena cava; LK: left kidney; RA: renal artery; SMA: superior mesenteric artery.

The present study examined 42 cadavers, in two of which the TA was absent (Figure 1). The origin of the TA was studied in the remaining 40 cadavers. For convenience, they were grouped into four types depending on the level of their origins in relation to the renal and inferior mesenteric arteries (Figure 2, Table 1):

**Table 1:** Classification of the testicular artery based on origin.

**Type**	**Right side (n = 40)**	**Left side (n = 40)**
I	36 (90%)	22 (55%)
II	0	6 (15%)
III	0	4 (10%)
IV	4 (10%)	8 (20%)

Type I: TA arising from the AA a little inferior to the RA (Normal pattern);

Type II: TA arising from the AA superior to the RA;

Type III: TA arising from the RA;

Type IV: TA arising from the AA at the level of the inferior mesenteric artery.

Although it has been stated that the TA normally arises a little inferior to the RA,^1^ in the current study, the distance between the RA and the TA was variable. The mean distances between the origin of the TA and the mid-point of the origin of the RA were 3.08 cm (minimum-1.1 cm & maximum-4.5 cm) and 3.47 cm (minimum-1.3 cm and maximum-5.9 cm), on the right and left sides respectively.

Additionally, we also noticed the variations described below. Interestingly, all the variations were observed on the left side. One case showed bilateral variations.

**Figure 3:**
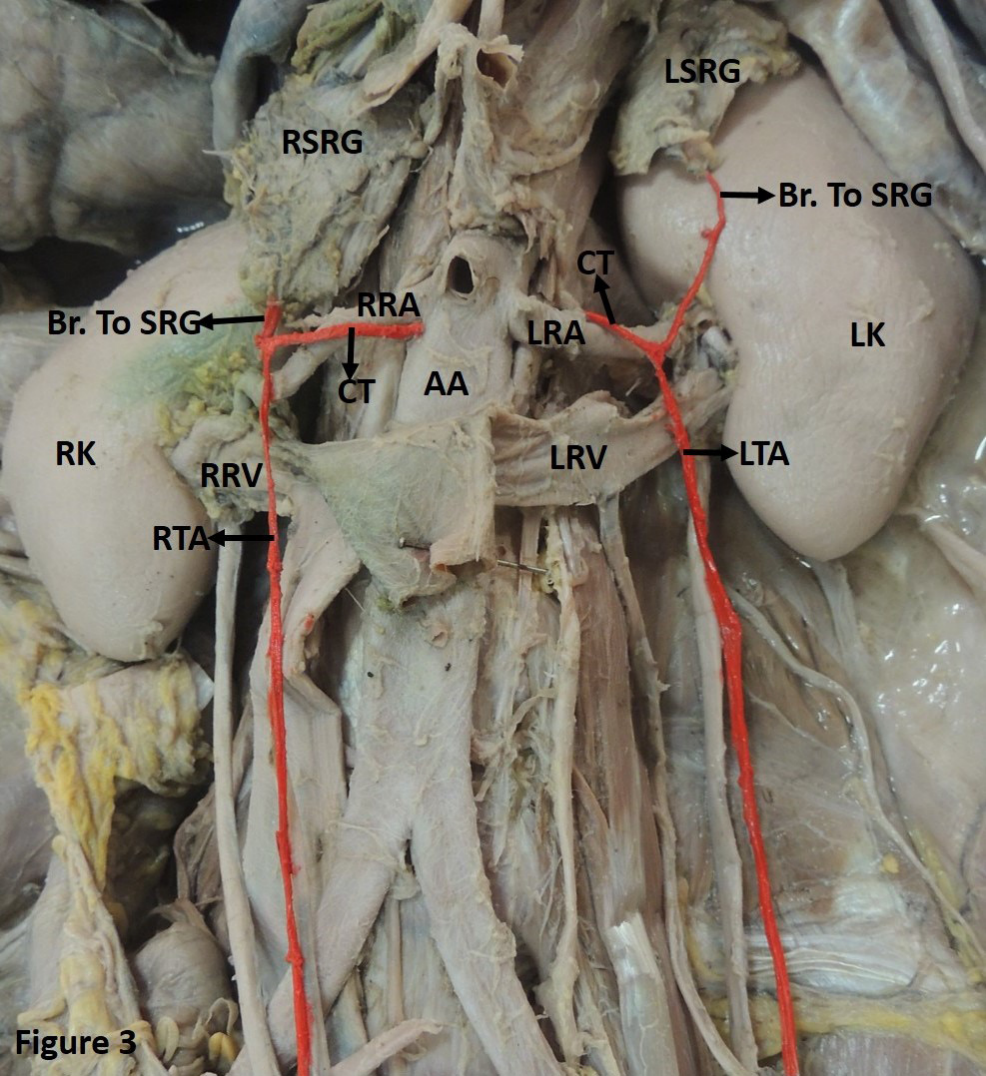
Showing a common arterial trunk (CT) arising from the renal artery (LRA) and abdominal aorta (AA) on the left and right sides respectively. The CT then bifurcates to provide a suprarenal branch (Br. To SRG) and further continues as the testicular artery proper (TA). Both the TAs (LTA and RTA) are seen to cross the respective left and right renal arteries (LRA and RRA) and renal veins (LRV and RRV) in this process. LSRG and RSRG: left and right suprarenal gland; LK and RK: left and right kidney.

Case 1: A common arterial trunk was seen to arise bilaterally. The left common trunk was observed to arise from the RA while the right originated from the AA, close to the origin of the RA (0.3 mm from the mid-point of the RA). Both the trunks then bifurcated to provide a suprarenal branch and further continued as the TA proper. The TA proper crossed the renal artery and vein bilaterally in this process (Figure 3). This finding was observed in a single case.

**Figure 4:**
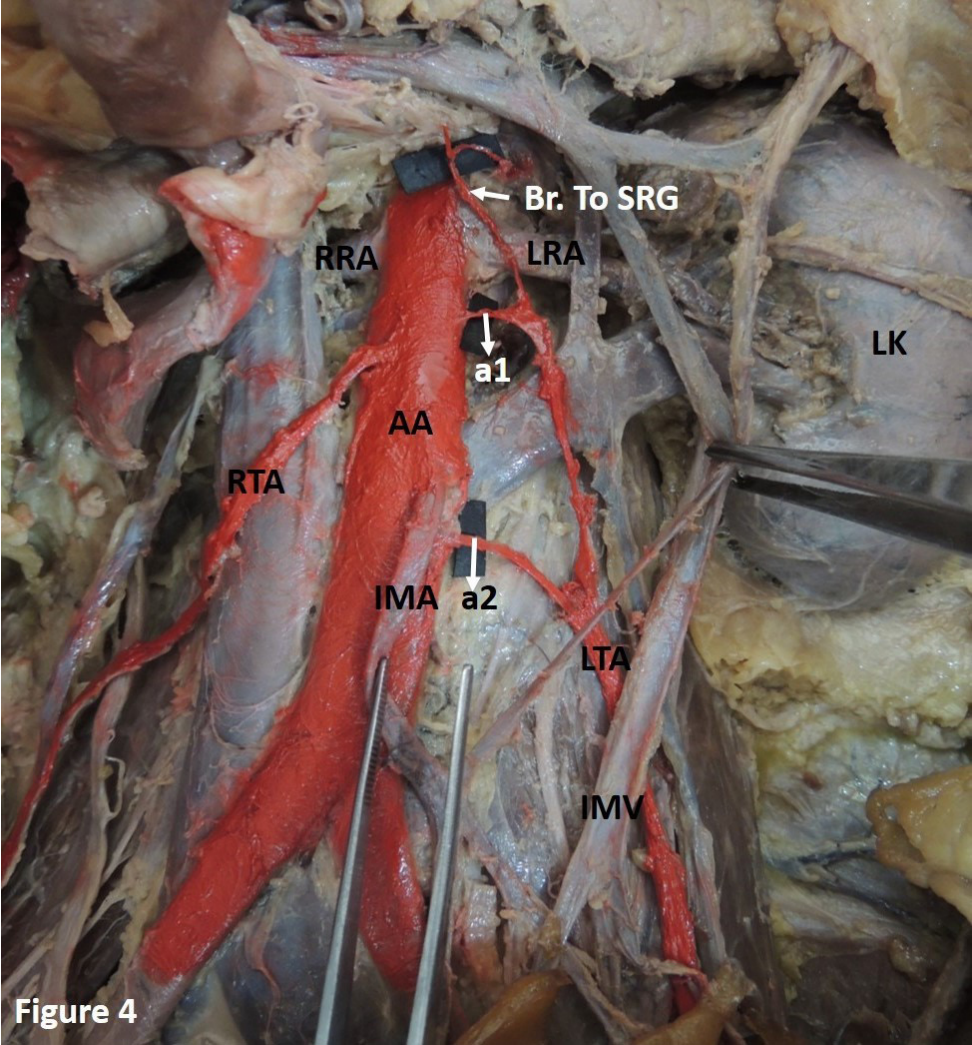
Showing the formation of the left testicular artery (LTA) by the union of two arterial twigs (a1 and a2) arising separately from the abdominal aorta. The upper arterial twig (a1) also provides a branch to the suprarenal gland (Br. to SRG) and the overlying connective tissue. AA: abdominal aorta; IMA: inferior mesenteric artery; IMV: inferior mesenteric vein; LK: left kidney; LRA and RRA: left and right renal artery; LTA and RTA: left and right testicular artery.

Case 2: The left TA was formed by the union of two arterial twigs arising separately from the AA at distances of 1.3 cm and 5.1 cm (at the level of the inferior mesenteric artery) from the mid-point of the origin of the left RA respectively. The upper arterial twig also provided a branch to the suprarenal gland and the overlying connective tissue (Figure 4). This was observed in a single case.

**Figure 5:**
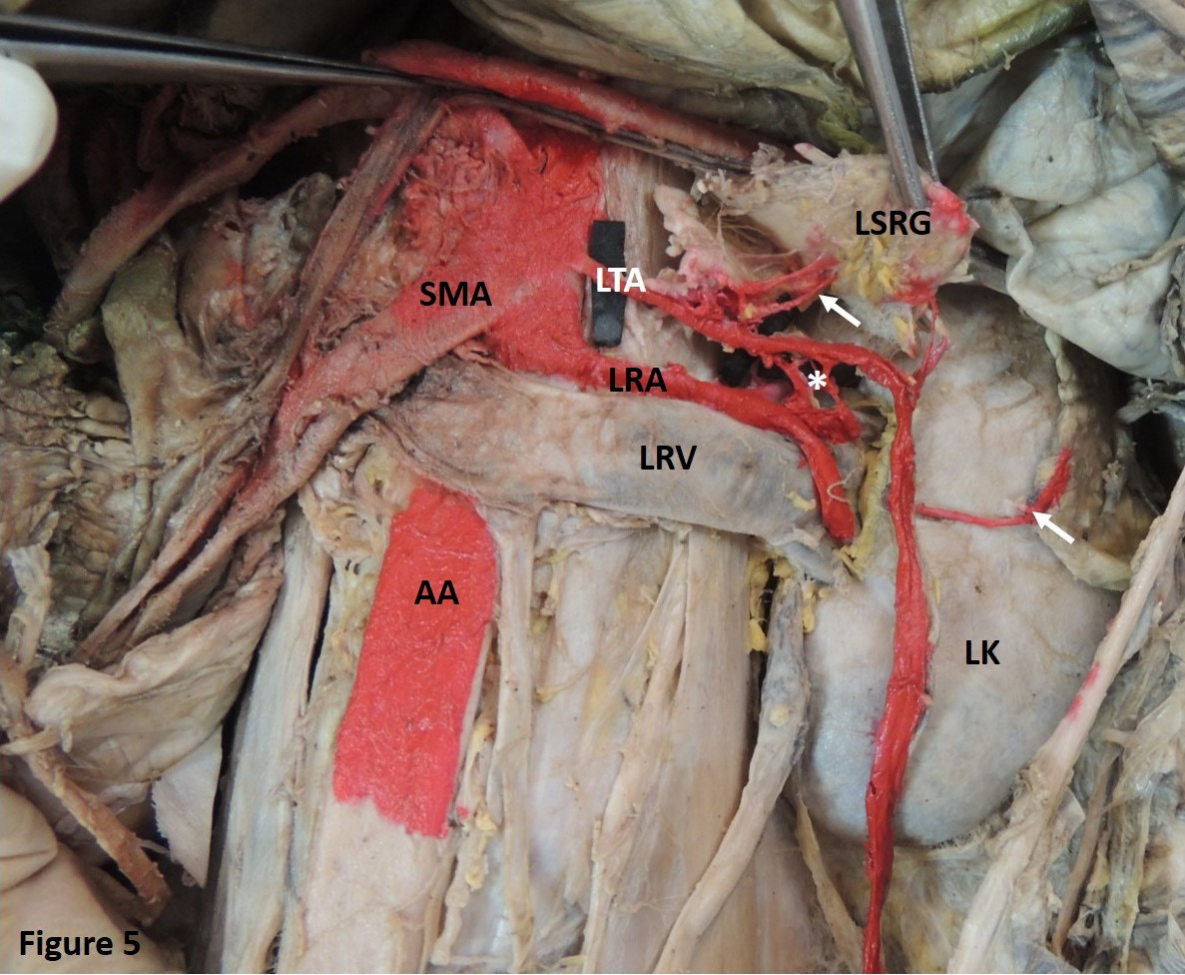
Showing the left testicular artery (LTA) arising from the abdominal aorta (AA) at the level of the superior mesenteric artery (SMA) and above the left renal artery (LRA). LTA provides multiple arterial twigs to the left suprarenal gland (LSRG) (Indicated by arrow). It also provides communicating branches to the LRA (Indicated by*). The LTA then descends superficial to the left kidney (LK). LRV, left renal vein.

Case 3: The left TA was seen to arise from the AA at the level of the superior mesenteric artery and above the RA. It extended laterally and provided multiple arterial twigs to the suprarenal gland. Communicating branches to the RA from the TA were also observed. At the hilum of the left kidney, the artery arched and descended superficial to the kidney and then lateral to the ureter in the posterior abdominal wall (Figure 5). This variation was observed in a single case.

**Figure 6:**
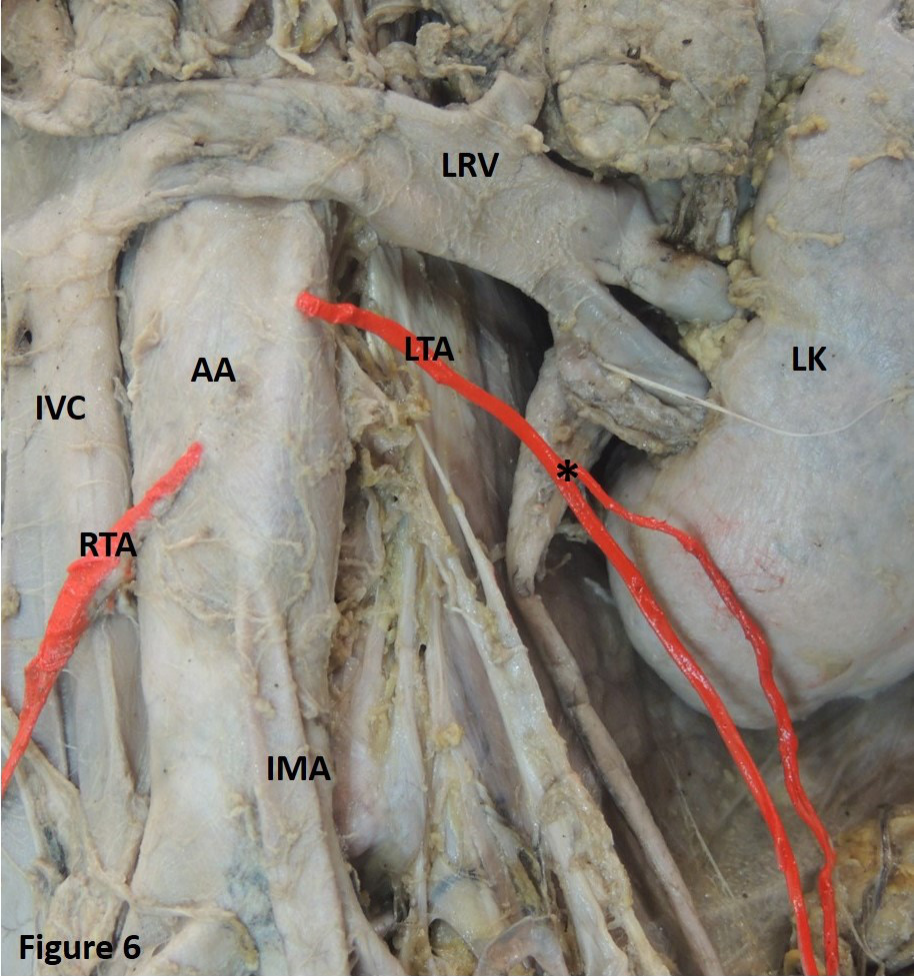
Showing duplication in branching of the left testicular artery (LTA) (Indicated by*). AA: abdominal aorta; LK: left kidney; LRV: left renal vein; RTA: right testicular artery; IMA: inferior mesenteric artery; IVC: inferior vena cava.

Case 4: The left TA showed duplication by branching. The artery had a normal origin and later divided into two. Both arterial twigs then traversed the inguinal canal (Figure 6). This finding was observed in two cases.

## DISCUSSION

As observed in the current study, and reported by many authors, the origin of the TA is variable.^4^^-^ 15 However, a classification based on the variable origin of the TA is seldom reported in the available literature. Although in the past authors have attempted to classify the TA based on its relationship with the renal vein, stressing its applied significance,^16^ this classification is not applicable to the different variations in the origin of the TA. As observed in the present study, the TA may emerge from above, i.e., at the level of the superior mesenteric artery, or from below, i.e., at the level of the inferior mesenteric artery, which cannot be described by the above-mentioned classification. Therefore, to facilitate understanding of the variations of the TA, the present study includes an attempt to classify the TA based on its origin.

The available literature indicates that the TA arises a little inferior to the RA.^1^ While the anatomy of the TA is relatively constant,^17^^,^ 18 occasional variations have been observed.^19^^,^ 20 In 5-20% of cases the origin of the TA is higher and in 5-6% it can arise from the main or accessory RA, as has been reported in previous studies.^4^^,^ 5 ^,^ 18 ^-^ 21 However, there could be a large range of levels of origin of the TA. Furthermore, the exact distance between the origin of the TA and the RA has not been explored previously. The present study has therefore attempted to provide this information. This is helpful to clinicians for locating the TA.

The TA usually arises from the AA inferior to the renal vein. Studies have also reported the TA arising posterior or superior to the renal vein. In 15% of cases, the TA arises from the RA, from branches of the RA, or even from a supernumerary RA. There are also cases in which the right TA emerges from the RA and the left from the AA, or vice-versa. The TA can even arise from an accessory RA, as reported previously.^22^ Rarely, the TA can originate from the suprarenal, phrenic, superior mesenteric, lumbar, common iliac, or internal iliac arteries.^2^ In the present study, the TA was observed to emerge either from the AA or from the RA and no other aberrant origins were observed. The TA can originate from the main or accessory RA, when it is termed the aberrant gonadal artery.^13^ Ambos et al. stated that this gonadal artery could provide a collateral supply to the kidney through the capsular arteries in situations such as RA stenosis and renal tumors.^23^ In one of the cases observed in the present study, the TA was found to provide communicating branches to the RA (Figure 5).

The TA can pass as an arched artery in front of the renal vein, as observed in previous studies.^2^^-^ 4 This artery has been named the artery of Luschka.^2^ In such conditions, the artery may get compressed leading to testicular degeneration.^24^ The present study identified a TA arching over both the renal artery and the vein (Figure 3). In another case, the TA was seen to arch over the kidney before descending to follow its usual course (Figure 5).

Further, in the present study, the TA was observed providing multiple arterial twigs to the suprarenal gland. In this case, the TA would have provided the sole supply to the suprarenal gland. The TA providing communicating branches to the RA is a rare finding that has seldom been reported (Figure 5). A common arterial trunk was also observed emerging from the AA, bifurcating to provide suprarenal and testicular branches (Figure 3). Jyotsna et al. made similar observations.^25^ Brohi et al. described a single case of high origin of the left TA providing a suprarenal branch.^7^ A similar finding was reported by Shinohara et al.^26^ Ondergolu et al.,^27^ identified a right TA providing inferior phrenic and superior suprarenal arteries.^27^ Knowledge of such variations could prove useful in management of spontaneous retroperitoneal hemorrhage resulting from adrenal artery aneurysm. It is also imperative during laparoscopic adrenalectomy, since it would affect the surgeon’s orientation.^25^

In another case, the TA was formed by the union of two arterial twigs arising at different levels from the AA (Figure 4). The TA emerging from the AA in the form of two or three roots and later merging into one has been reported before. In such instances, they normally arise between the levels of the first and the third lumbar vertebrae.^2^

The TA may be doubled, tripled, or even quadrupled.^4^ Authors have also observed a double TA arising bilaterally from the AA.^25^ In our study, we observed a case in which a single trunk of the TA later doubled and then continued into the inguinal canal (Figure 6).

Our review of the literature suggested that most of the TA variations were on the right side.^15^^,^ 28 However, in the current study all the variations were on the left side. Additionally, a common trunk providing suprarenal and testicular twigs was observed bilaterally. This is another unique feature of our study.

In the present study, the TA was also found to be absent (Figure 1). Absence of the TA has been reported previously. Authors explained this fact by stating that if one or both TAs are absent, the testes would be supplied by branches from the vesical or prostatic arteries.^2^

The variability of the TA is attributed to its development. As explained previously, there are approximately nine lateral mesonephric arteries, which are grouped into three sets: cranial, middle, and caudal. These arteries generally disappear caudally, except those supplying the kidneys, gonads, and suprarenal glands. One of the caudal arteries normally persists and forms the definitive gonadal artery.^29^ A persistent cranial lateral mesonephric artery might have resulted in the high origin of the gonadal artery seen in the present study. It might also have been responsible for a common arterial twig supplying the suprarenal gland and the gonad, as identified in the present study. Arching of the TA over the left kidney can also be explained by the higher origin. The low origin of the TA might be due to persistence of the lower twigs of the caudal group of the mesonephric arteries. Certain factors like growth, hemodynamics, genetic factors, and teratogenicity of chemical reagents may also influence the aberrant origin of arteries.^30^

With the advent of novel surgical techniques within the abdominal cavity, the anatomy of the TA has gained greater significance. The TA must be well preserved to avoid the possible complications resulting from damage to it.^31^ This study therefore provides insight into variations in the testicular artery and offers a classification which could help surgeons during a variety of procedures on the male abdomen and pelvis.
